# Education as a Sociological Factor

**Published:** 1902-10

**Authors:** George H. Price

**Affiliations:** Nashville, Tenn.


ATLANTA

### Journal-Record of Medicine.

Successor to Atlanta Medical and Surgical Journal, Established 1855, and Southern Medical Record, Established 1870.

VOL. IV. OCTOBER, 1902. NO. 7.

BERNARD WOLFF, M.D., EDITOR, Nos. 319-20 Prudential.

Published Monthly.

M. B. HUTCHINS, M.D., BUSINESS MANAGER, No. 64 Marietta St.

### ORIGINAL COMMUNICATIONS.

### EDUCATION AS A SOCIOLOGICAL FACTOR.*

BY GEORGE H. PRICE, M.D.,
NASHVILLE, TENN.

It would be impossible, in the brief time given to papers presented for discussion, to attempt a complete résumé and elaboration of all that is implied in this comprehensive question; therefore, it will be my aim to draw attention to some of the leading features involved, with the hope of directing attention along educational lines.

I believe that all of us recognize the fact that education is one of the basic factors in sociology. The dissemination of knowledge is one of the prime motives of the work which this Society has undertaken, and upon it is largely dependent the success which you hope to attain. We cannot hope to impress upon those who have not stopped to think of sociological problems their importance,

*Read before the Georgia Sociological Society, Atlanta, June, 1902.

for the reason than even the more thoughtful are not in possession of enough information upon the problems involved to enable them to fully grasp their significance; consequently they are not in a position t) express a competent opinion or advance a reasonable working-plan upon which to proceed. This we can at once see is due to ignorance—ignorance born of a lack of observation of the minute detail of the sociologic conditions which surround us; though we may have been impressed with the fact that the general conditions of the body politic, in its broadest acceptation, are below the standard which should exist in any given community. The investigation of any complex question, presenting many intricate phases, is no easy undertaking, and can only be entrusted to those who have acquired by training and experience the peculiar faculty of drawing conclusions from facts presented; that is, investigators who can trace a cause from an effect, or who can forecast an effect in a given cause. If this be true, then we must conclude that the promoters of social science must be men of education. We often hear the expression, "The public must be educated up to the point where it can appreciate its opportunities, its needs, or its real condition," and this is very true. To educate the public up to any given condition, project, enterprise, opportunity or social plane will depend primarily upon the mental grasp of the public as a whole. This we often see, as is illustrated in music, art, science, literature, public health and new features in good government, looking to social reform. Some communities are lovers of good music, while others have a low standard and only appreciate that which appeals strongly to the lowest ideals. The love of music of high type is only engendered in the public mind or ear, as a whole, by the oft-repeated rendition of high-class music by trained musicians. The music of Wagner, Bach, Beethoven and such composers often fails to arouse any enthusiasm in an audience, as a whole, because the individuals composing the audience are not prepared to follow the theme of the composer, while the rendition of the compositions of what are called popular composers, such as Straus, Sousa and lesser lights call forth prolonged applause, and should the band or orchestra respond to the encore with "Old Folks at Home," "Dixie," or

"The Star-Spangled Banner," the audience rises as one man and cheers to the echo.

The same conditions, generally speaking, apply to art, science, literature and public health. All of us are more or less familiar with the difficulties which have confronted the authorities, both state, county and municipal, when they have been called upon to combat some threatening epidemic, which, specterlike, stood upon our borders with the uncorked vial of wrath, from which was beginning to drop the deadly germs of disease. The experiences of many of us have long since taught us that it is too late then to educate the public, because the public is panic-stricken, but when the panic subsides the education is begun, and experience again teaches us what a paying investment these educational efforts are. Thus we see that any important step by which it is hoped to elevate the general public above the plane on which it has been moving is often fraught with difficulties and must experience delays, many times dangerous to the undertaking, which delays are primarily due to ignorance or lack of education. Nor can these steps in public improvement be suddenly proposed and speedily accomplished, but rather they are slowly evolved and oftentimes more slowly brought to full maturity, while the public is being educated up to them.

Now these generalizations are only to show that every important move for the public good must pass through a process of evolution in the public mind, during which time it is being considered, studied, weighed and discussed by the public. In other words, the public is being trained, or educated, by the more advanced thinkers, the leaders, who, by reason of their advantages, due in a large measure to their educational acquirements, are in a position to advise, to encourage, to lead and direct public thought and mould public opinion. When, therefore, we closely inspect and analyze any upward movement, we must necessarily look into the influences which underlie it, and here we find that education is the motive power or factor of most importance. Again, if we analyze any marked and pronounced movement which threatens the existing social conditions of a community, large or small, we will find ignorance as its prime factor. If, then, this be true, would it not be well to lay the foundation in the public mind upon which can be built that superstructure which is so essential to the public good ? The answer to this question is obvious. But then the further question arises, how is this to be accomplished ? Shall we wait until the public is confronted by conditions which threaten the social equilibrium before the educational effort is made, or shall we begin this process at a time when the public elements are in a formative state, plastic, so to speak, easily impressed and easily moulded ? I hardly think that there are even a few here who would advise waiting, for all are convinced that "as the twig is bent, so the tree is inclined." In view of these facts, I am sure that we can safely assume that previous preparation, that is, education, will prove a most valuable adjunct in meeting the requirements under progressive sociologic conditions. The reasons herewith advanced in favor of education as a factor in social problems are those which are most apparent, even to the casual observer, nor are they new, but in fact old, for the founders of our government saw, in the dim penumbra of coming events, the possibilities of these questions and sought to impress the same upon those who were to come after them by suggesting that the final stability of our institutions was dependent upon the educating of the masses. This prophetic insight into the future of our country was not heeded as it should have been, and consequently education languished for a time, but now gives promise of better things for the future. These are what we might term general, social or political reasons for education.

There are other reasons for stressing this important aid to sociologic science; and here we are brought face to face with some striking and convincing results obtained by investigators along the lines of practical physiology. Microscopic investigations of the human embryo have revealed the fact that the number of cells in the central nervous system is not increased after the third month of fetal life, yet the brain increases in size, and the capabilities of it are immensely increased, as we all know. This being true, the question arises, how then does the brain grow and how are the mental faculties expanded ? The answer is obvious; the individual morphological elements present in the original mass, in a dormant state, begin to take on developmental changes to meet the ever-increasing demands made by the growth of the organism as a whole; for the brain is the great presiding genius, the directing power of the organism. We see from this that there are numerous cells which are not physiologically active from the beginning of the life of the individual, but are brought successively into physiological relation with those already correlated and performing their peculiar functions. In order that any nerve-cell may take part in the general functions of the nervous system, it must not only increase in size, which increase is many thousands of times that of the original germinal cell, but must also throw out its axon or axons and dendrites, thus becoming a real neuron, establishing communication with near or remote cells and becoming a real physiological factor, capable of performing its peculiar functions. If these cells should never develop beyond their embryonic stage, then, as a matter of course, they would never enter into or take part in the general physiological activities or processes of the nervous system; that is, they would be functionless. If they remain functionless, the brain, as a consequence, would remain undeveloped to its full capacity, hence it would fail to measure up to its full standard, both in receiving and giving-out impulses, which, in its final analysis, means that the individual possessing such a brain would fall below the standard to which that individual should attain. This being true, we naturally conclude that the functional possibilities of the brain will depend upon the evolution of the embryonic brain cells into the fully matured cell. This evolution is dependent upon the demand made upon the embryonic cell mass for an increased number of developed cells to perform an increased amount of work. This increased demand, acting through the processes of nutrition, calls into action those changes which pass from polarization of the embryonic cell to the development of axon and dendrites, whereby the simple cell becomes the fully developed neuron, capable of performing the physiological function demanded of it. There is no absolute or fixed standard by which to accurately measure brain capacity, but there is an approximate method, which I think offers a good working basis, namely, that of the weight of the brain mass. The evidence presented in Marshall's tables, based upon Boyd's records of brain-weights, is quite interesting. (American Text-

Book of Physiology.) From these tables it is shown that the brain-weight is greatest between the ages of twenty and forty years, after which time it recedes, reaching its low mark between seventy and ninety years. As to the interpretation of weight, I quote the following from American Text-Book of Physiology:

"Assuming as the simplest case that the number of the nerve-elements composing a given portion of the central system is *constant* within the limits of the same species, then the differences in the weight of these portions in different individuals imply variations in the size of the component cells. The significance of variations in the size of the nerve-elements must be, primarily, that the larger the cells, and especially the larger the cell bodies, the greater the mass of cell-substance ready at any moment to undergo chemical change leading to the release of energy, and the more numerous the probable connections. . . . The loss of weight in advanced years appears to be due to a general atrophy of the nerve-elements."

The weights recorded in the tables above referred to are of brains taken from the average patients of the hospitals, and as a matter of course represent the lower stratum of social life, where the individuals have not had the best environment. In this connection I desire to quote again from the very reliable source above referred to on the effect of social environment, in which are given the conclusions of Donaldson, on the subject of the Growth of the Brain, 1895. Under this head, Social Environment, it says: It is not to be expected that the weight of the brain among the least favored classes of any community will be the same as that of those who, during the years of growth, are under favorable conditions. All extensive series of observations which we possess relate to the least favored social classes, and hence it is not improbable that the figures in the foregoing tables, which are based on data obtained mainly at the Marylebone workhouse in London, are decidedly below those which would be obtained from the more fortunate classes in the same community. We have a list of brain-weights which contains the records for a number of men of acknowledged eminence, and also for others who obtained recognition as able persons, without being exceptionally remarkable. This list shows the persons thus selected to have brains on an average heavier than the usual hospital subject.

These are significant facts and are surely worth the serious consideration of those who are endeavoring to solve those problems relating to the uplifting of their fellow-men. From this record of observations made by competent investigators, there is conclusive evidence that as social environment improves, brain-weight improves, and as brain-weight improves, the social grade of the individual improves. Social environment of high grade, which is based upon culture and refinement in their broadest and most comprehensive sense, is based primarily upon education, which we all recognize as the fundamental factor.

In addition to these observations upon brain-weight, the growth of the brain at different ages presents some very interesting features, which might be well worth our consideration. Investigations of Vierodt and Bischoff have demonstrated that at birth the brain is only about one-third the weight of the adult brain. The most rapid increase is during the first year; from the first up to and including the seventh or eighth year the growth is still very rapid, but that from this time on the growth is slow, though it gradually increases up to the twenty-fifth year, then with slight fluctuations to the thirty-fifth year, when a slight but gradual increase is maintained for twenty years, or up to the fifty-fifth year, when the decline sets in and continues until the end of life. This is the line followed in the average brain, or what might be termed the general social level. However, in the brains of eminent men the increase of growth continues up to the sixty-fifth year, when the decrease becomes apparent. The study of this graphic scheme of curves, as given by Vierodt and Bischoff, will prove most interesting. It will be observed that the brain at the end of the *first year* is nearly 78 per cent. of the adult brain; at the end of the fourth year the brain is 95 per cent. of the adult brain, and between the seventh and eighth years it has reached the adult average. During these first few years of brain-growth, say up to the fourth year, the embryonic cells are rapidly becoming mature, but this rapid growth is, perhaps, largely confined to groups of cells which are especially interested in the control of bodily movements, without the intervention of the finer distinctions of reason. In other words, the condition is one which approaches automatism. This we often see in the spontaneous aimless movements of young children, who seem to be possessed with the demon of perpetual motion. This is, perhaps, a period previous to the full development of the associating tracts whereby this spontaneous aimless discharge of motor impulses is brought under the controlling influence of what is termed reason. From the fourth to the eighth year the growth is still very marked. During this time the embryonic cells are not only developing into true neurons, but are also establishing collateral connections with other cells, and the individual is becoming more and more amenable to reason. In other words, the child is now reaching a point where it can begin to take on an education. A study of these facts would seem to indicate that, during the earlier period of this process of brain-growth, that the methods of teaching or impressing the child-brain should be by those channels which appeal to the special senses, and not by abstract reasoning, as the physiological basis of the latter is not yet sufficiently developed to warrant it. Upon these facts depends the success of the Kindergarten plan, now so very popular.

The most marked variations of brain-growth are noted between the twelfth and sixteenth years, and is a most important period in the life-history of all individuals, as we understand, since about this time cluster some of the most important physiological changes. From this time on the changes are not so marked, but there is gradual brain-growth up to about the fifty-fifth year in males, and the forty-fifth in females. This is significant, for it at once suggests, as we know to be the case, that the average man is more given to active brain-work than the average woman; hence, there would be an increase in brain-weight unaccounted for in the greater body-weight alone. Again, these observations show that where the individual had obtained eminence the brain-growth continued up to the sixty-fifth year, by reason of the fact that the brain was kept in training, or that the educational influence was continued up to this time.

It is a well-known-fact, to the most casual observer, that systems other than the central nervous system, can only be developed by training or educating them, and that there is a time limit, before which such training is not advisable, except in the lightest form, and also one after which it is almost impossible to accomplish much. We all know the history of the athlete in and out of training, or the result of training too young, or waiting too late to train. The expression "You can't teach an old dog new tricks" is a homely summing up of a physiological proposition. The clay in the hand of the potter is only susceptible of being moulded and fashioned into beautiful forms while it is plastic; so also we find in all the arts, sciences, and education, that there is a time when conditions are favorable, and that this time and these conditions must be taken advantage of in order to obtain the best results.

In view of these facts, it seems to me that we can draw some important and profitable lessons therefrom, and apply them to the question we have under consideration. In the first place, it is evident, from general considerations, that the masses can be elevated to a higher standard or plane by placing in their reach those influences which have an elevating tendency upon the people as a whole. It seems also evident that these influences act by educating the masses up to those standards or levels which are usually enjoyed by those of the more fortunate classes. It is also evident, from a scientific standpoint, that, in the ultimate analysis of social questions within the limits of the same species, the physiological basis upon which the social fabric is founded, is practically the same for all individuals. Again, that there is a time in the history of each individual when this physiological basis, or factor, is more susceptible of development than at other times, and that this time is quite extended. These things being true, it seems that the failure of the masses to rise to the higher planes of social life is dependent upon the failure of the individuals to develop along those lines which are physiologically common to the classes, as well as to the masses.

We have seen that this development can only come through social environment, which plays such an important part in determining the trend or bent of the individual along a higher or lower social plane. Thus we see that the social environment comes to mean much in this question, and as it is either progressive or retrogressive, so will be its influence. Progressive social environment can not be attained or maintained without the elevating influences of culture and refinement, and these are only brought about, nurtured, matured, and made permanent and lasting by and through education. But education can only be propagated by the continuous and urgent effort of those who have education, for no one can impart that which he has not. Again, to be effective, the effort to influence must be applied at the proper time ; that is, during that period when the brain, the material organ of the mind, is in the most receptive condition. This influence which brings about those progressive changes in the embryonic cells of the brain-mass, which calls into physiological activity a greater number of these cells, and causes them to establish a greater number of collateral connections between other cells and groups of cells, both near and remote, than would otherwise be brought into these vital relations, under ordinary and undirected effort, is that which, upon final analysis, is found to be education in its truest sense.

Thus we see that education is but the ideal evolution along physiological lines already indicated in nature's process of development of that great organ, the central nervous system, the master tissue, which presides over all the functions of the body, originating and directing all those complex ideas, feelings, sentiments, expressions and movements whereby man is distinguished from the lower forms of animal life.

The object of this paper is not to develop any system of education, but rather to call attention to the fact that there are reasons—political, social and physiological—for this body insisting upon the State making those efforts which are necessary to place in the hands of those coming after us every opportunity for gaining an education. I am convinced that it would be impossible for the State to undertake the higher education of the masses, for this is both impractical and unnecessary, but then the State should undertake their fundamental education, and the effort should be made at that time when the organ to be developed is in its most receptive and retentive condition, namely, from eight to sixteen years.

Another thing to which I desire to call attention is that education furnished by the State should be along more practical lines, for the tendency of the times is toward the practical, and education should be directed along the same lines.

Allow me to say, in conclusion, that I hope that some one who is familiar with the educational problem, will perfect and propose a plan embracing both the fundamental and the practical features for state educational institutions, for upon education depends immediately and remotely the stability of our institutions, and the social elevation of the people.

